# Mapping knowledge landscapes and emerging trends of the links between osteoarthritis and osteoporosis: A bibliometric analysis

**DOI:** 10.3389/fpubh.2022.1019691

**Published:** 2022-12-19

**Authors:** Xin Wan, Xuefei Wang, Ran Pang, Chunlei Xu, Wei Shi, Huafeng Zhang, Hui Li, Zhijun Li

**Affiliations:** ^1^Department of Orthopedics, Tianjin Medical University General Hospital, Tianjin, China; ^2^The First Surgical Department of Breast Cancer, Tianjin Medical University Cancer Institute and Hospital, Tianjin, China; ^3^Department of Orthopedics, Tianjin Hospital of ITCWM Nankai Hospital, Tianjin, China

**Keywords:** osteoarthritis, osteoporosis, bibliometric analysis, visualization, CiteSpace, VOSviewer

## Abstract

**Background:**

Osteoarthritis has the characteristics of degenerative changes in articular cartilage and secondary osteoporosis, and it is a common chronic irreversible joint disease. In addition to affecting articular cartilage, subchondral bone, joint capsule and synovial membrane also undergo pathological changes during the development of the disease. Multiple studies have revealed that patients with osteoarthritis were found to have a significantly increased risk of osteoporosis, which also contributes to the progression of osteoarthritis. However, in the current existing studies, we found that no scholars have used bibliometric analysis in the study of the relationship between osteoarthritis and osteoporosis. From the perspective of bibliometrics, this study summarizes in detail the degree of cooperation between countries, research institutions, authors, and related journals in the field of osteoarthritis and osteoporosis research and their respective influence. In this way, the evolution of knowledge structure, the change of research focus and the hot topics with research potential in the future can be further visualized and analyzed.

**Methods:**

Search the Web of Science core collection in Science Citation Index Expanded for articles and reviews of research on osteoarthritis and osteoporosis from 1998 to 2021. Bibliometric tools such as VOSviewer, CiteSpace, were be frequently used in our study. They are mainly used to analyze collaborations between countries, research institutions, and publication authors. Meantime, co-citation analysis of journals, co-occurrence analysis of keywords and subject categories will also be reflected in the study.

**Results:**

According to the search strategy, 1,078 publications were included during the period 1998–2021. And the number of annual publications on the relationship between osteoarthritis and osteoporosis is on the rise. The United States has achieved the most and contributed the most in this field and the Boston University was the most prolific institution. For the statistical analysis of published publications, Reginster JY had the highest number of publications, while Felson DT had the highest co-citation frequency. Respectively, Osteoarthritis And Cartilage was the most productive journal in this area of research. The keywords “inflammation,” “expression,” and “mesenchymal stem cells” may also be the development trend and research hotspot of the future research direction in this field.

**Conclusions:**

In our study, the relationship between osteoarthritis and osteoporosis was analyzed by using literature measurement. These analysis results can lead researchers to learn more directly about the trend in this area and provide guidance for determining popular research directions.

## Introduction

Osteoarthritis (OA) is the most common degenerative joint disease. It may affect one or more joints at the same time, including the small joints of the hands and feet and the large joints such as the hips and knees (1). The initial lesion occurred in the cartilage, and then invaded the subchondral bone plate, the synovial membrane, and other periarticular tissues. Focal and erosive cartilage destruction, subchondral bone sclerosis, cystic changes, and compensatory osteophyte formation were observed. Its occurrence is mainly related to age factors, body weight, immune inflammation, sports injuries, fatigue use, and metabolic disorders in the internal environment, and genetic factors. OA affects more than 240 million people worldwide, 28% of whom are over 60 years of age. With aging, the incidence of numerous complications, including disability, reduced quality of life and death, is also on the rise (2). Until now, the treatment of OA has remained challenging, and its risk factors and pathophysiology are still evolving (3). On the other hand, according to the definition by the World Health Organization, Osteoporosis (OP) as a systemic metabolic disease, is characterized by reduced bone density and damage to bone micro-structures, so patients with OP generally have weak bones and are prone to fracture, affecting approximately 10 million Americans in the United States, More than 34 million patients have low bone mineral density, the incidence of OP is significantly increased in postmenopausal women (4, 5). It is similar to OA in that it also has a higher incidence and certain disabilities (6).

Most past studies have focused on the inverse relationship between the two diseases because they are thought to rarely coexist in a single individual (7, 8). But in recent years, many large sample multicentre clinical trials have focused on revealing the relationship between OA and OP. Excessive bone resorption is a significant sign of postmenopausal OP. But interestingly, excessive bone resorption is also seen in the early development of OA, while osteosis, subchondral bone sclerosis and osteophyte formation occur in the early and late stages of OA development (9). In addition to the overlap in disease progression, there was also substantial evidence that inflammation also influences the development of both OA and OP (10). Since the beginning of the new century, a lot of research has been conducted on the relationship between OA and OP, suggested that inflammation, foreign bodies and excessive mechanical load are common factors affecting the development of OA and OP. De Laet found that foreign bodies contributes to the development of OA and OP, but the mechanism of action is different. Obesity contributes to the progression of OA by adversely affecting the mechanical action of weight-bearing joints and specific adipose tissue-derived factors. In contrast, underweight or having a low BMI is a well-known risk factor for OP (11).

In view of the above aspects. The relationship between OA and OP has attracted the attention of many experts and scholars in this field. At the same time, as mentioned above, some scholars have noted that some common factors influence the development of the two diseases, so these insights may also contribute to the research and development of new therapeutic methods (10). On the other hand, with the deepening of research and the rapid in the number and rate of annual publications, for scholars in the field to keep up with the latest findings is difficult. Even in the fields, they have specialized in for many years, most of them only focus on the research from a single perspective. However, the global research trend of the relationship between the two has not been systematically studied and found. Such information is very meaningful but often ignored, such as the growth trend of the number of publications, the contribution of countries and regions, and the contribution of institutions and authors, which can intuitively present the hot spots in the future research field to the majority of scholars. Bibliometric analysis as the system and characteristics of literature research has been widely used in the process of qualitative analysis of scientific literature (12, 13), which has also become a popular method for us to obtain the above-mentioned parameters.

Bibliometrics is an interdisciplinary subject that uses mathematical and statistical methods to quantitatively analyze the knowledge carriers of research. It is a body of knowledge focused on quantification, integrating mathematics, statistics, and documentation. At present, bibliometrics analysts such as Vosviewer (14) and CiteSpace (15) are also working with the theoretical basis. These tools are also used in the bibliometric analysis of various disciplines, such as orthopedics (16), respiratory diseases (17), autoimmune diseases (18), COVID-19(19) and cancer (13). In addition to the field of medical health, bibliometrics is also used in the research of intelligent computing, cybernetics and other fields, and has a wide popularity in the process of scientific research activities (20–23). It is worth mentioning that bibliometrics is also widely used in service network and group decision making (24, 25). From our review and analysis of previous literatures, it is found that some scholars have published bibliometric studies only on OA or OP, on the basis of the above, scholars can grasp the relevant studies of OA or OP respectively, and they can have a clear understanding of the separate research focus and hot spot of the two diseases. But there has been no bibliometric study on the relationship between OA and OP up to the beginning of this study. Therefore, many scholars lack an overall view of the research on the relationship between OA and OP, and it may be difficult and vague to control the research direction of the relationship between the two, which will ultimately have a great impact on the quality of research results they may publish in the future. Hence our research is feasible and instructive, this study was designed to provide a comprehensive analysis of related publications published between 1998 and 2021, identify the change of research popularity in this field over time, determine the countries and institutions that have made major contributions to this field, the distribution of funding departments for this field, and explore relevant information such as authors and co-cited authors, journals and co-cited journals, and keywords. The above situation is visualized, which enables subsequent researchers to have a clear understanding of the main national institutions, authors and sources of relevant publications in this field, and speeds up the author's acquisition of key points. The above information can guide relevant researchers to conduct academic exchanges and discussions with countries, authoritative institutions and key scholars when they encounter doubts in the study of the relationship between OA and OP, and to cooperate with scholars from these institutions if necessary. I believe this will be of great help in producing high-quality research in these fields in the future. Obviously, this will also have clinical implications for future synergistic treatment of these two diseases.

## Materials and methods

### Data source and retrieval strategy

**Figure 1:**
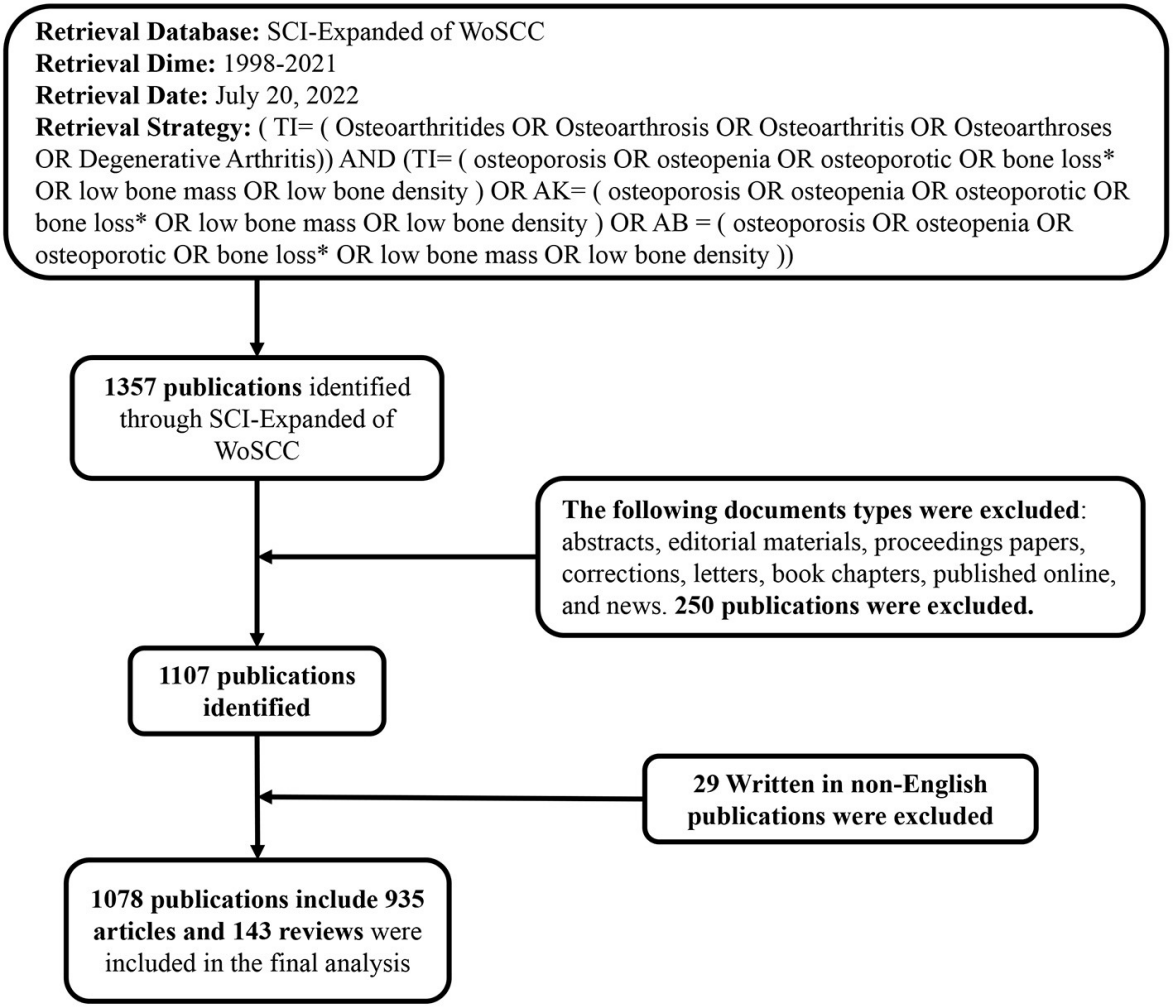
Flowchart for the selection of literature included in the study.

The Web of Science Core Collection (WoSCC) is one of the important data sources for obtaining authoritative academic information around the world. It contains more than 20,000 authoritative and high-impact academic journals, more than 200,000 conference proceedings, and abstracts of more than 100,000 scientific and technological books, The Science Citation Index Expanded (SCI-Expanded) of the WoSCC has been used in the scientometric analysis because this platform provides the most comprehensive information needed for bibliometric analysis tools (26). To avoid bias due to daily database updates, our study retrieved and downloaded all literature identified from the SCI-Expanded of WoSCC from January 1, 1998 to December 31, 2021. The retrieval style of literature retrieval was determined by reference to the results of previous studies. Therefore, OA and OP were the main research object in our study. In order to obtain accurate retrieval results, we have developed a good search strategy (Figure 1). A total of 1,357 articles were retrieved, including ten types of documents. There were 972 articles, accounting for 71.629%, it identified that articles were the most common type of literatures, and there were 148 reviews accounting for 10.906%. The other eight types documents include abstracts (210), editorial materials (18), proceedings papers (11), corrections (4), letters (4), book chapters (1), published online (1), and news (1) as shown in Table 1. We excluded eight document types and non-English literature. Two researchers (XW and XF-W) searched the raw data independently, then the two researchers discussed the different opinions in the study and reached an agreement of 0.95 (27). Ultimately, we obtained 1,090 valid documents as the final data source, the detailed filtering process is shown in Figure 1, and selected the form of “full records and cited references” to export as plain text documents for further analysis. In addition, due to the limitation of CiteSpace, all text files analyzed by CiteSpace must be named starting with “download.”

**Table 1:** Document types of the publications.

**Rank**	**Document type**	**Record number**	**% of 1,357**
1	Articles	972	71.629
2	Meeting abstracts	210	15.475
3	Review articles	148	10.906
4	Editorial materials	18	1.326
5	Proceedings papers	11	0.811
6	Corrections	4	0.295
7	Letters	4	0.295
8	Book chapters	1	0.074
9	Published online	1	0.074
10	News	1	0.074

### Data extraction and analysis

The datasets were imported into CiteSpace software first to remove duplicate data. Then relevant data including publication frequency and citation frequency, distribution of countries and institutions, all authors, funding agencies, publications, and journals, cited articles, and keywords are extracted. The journal information including impact factor (JIF) and Quartile in category (Q1, Q2, Q3, and Q4) was obtained from the 2021 Journal Citation Reports. Statistical analysis was performed using SPSS20 (IBM, USA) and Microsoft Excel 2019 (Microsoft Corporation, Redmond, WA, USA).

Bibliometric and visualization analyses were conducted by three bibliometric tools. CiteSpace, free Java-based software developed by Chen (28), was visual analysis tool developed gradually in the context of scientometrics and data visualization. Because it can visually and intuitively show the distribution of knowledge to researchers. In the network maps, nodes represent items such as countries, institutions, authors, and so on. The size of the nodes indicates a quantity, and the color rings indicate different years, respectively. The lines between the nodes reflect the cooperation or co-citation relationships of items. In this study, CiteSpace was used to make visual analysis of countries and institutions, analysis of the authors and co-cited authors, the dual-map overlay of journals, and visual analysis of co-citation and clustering network.

VOSviewer, another bibliometric software developed by Professor Van Eck and Waltman (14), VOSviewer can construct and visualize the relationship of “network data” (mainly literature knowledge units), and realize the drawing of a scientific knowledge graph to show the structure, evolution, cooperation and other relations of the knowledge domain. Its main characteristic is strong visualization display ability, suitable for large sample data analysis. In addition, VOSviewer could provide three types of network maps (21, 23), including the network visualization map, the overlay visualization map, and the density visualization map, which are used to present network visualization map of journals and co-cited journals as well as a visual analysis of keyword co-occurrence (29, 30). Moreover, We also used an online bibliometric platform (https://bibliometric.com/) for the country collaboration analysis.

One of the main components of Microsoft Office, Excel is a spreadsheet program [a software program that performs numerical and budgetary calculations]. It was the first Office component. Excel has built-in functions that can classify, sort and even chart large amounts of data. We used Excel to calculate the number of publications and citation frequency every year, and made the results into statistical charts to present. Meanwhile, all the tables in this study were also completed by Excel.

## Results

### Visualization of publication and citation trends

**Figure 2:**
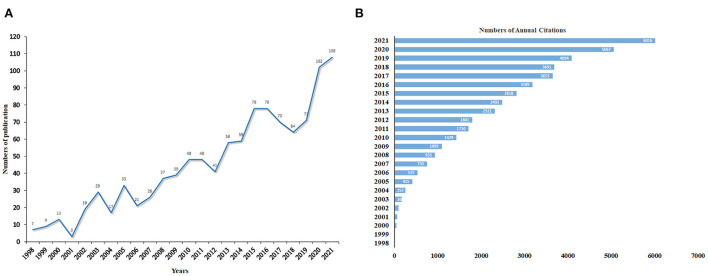
**(A)** The Number of annual publications examining the relationship between OA and OP from 1998 to 2021. **(B)** The citation frequency.

According to the retrieval strategy, from 1998 to 2021, there were 1078 publications on the relationship between OA and OP, including 935 original articles and 143 reviews. The changes in the annual number of published articles and the frequency of cited articles reflect the development trend and the degree of research focus in this research field (31). As presented in Figure 2A, since 1998, the annual publications generally showed a growth trend, and 2020 was the fastest growth year, with a growth rate of 43.66%, and the publication reached its peak in 2021, with a total of 108 publications. It accounted for 10.19% of the total publications. There were small declines in 2001, 2004, 2006, 2012, and from 2017 to 2018. In addition, Figure 2B showed that the citation frequency also showed a continuous upward trend from 1998 to 2021. These publications have accumulated 45,768 citations, excluding the number of self-citations, there are still 42,399 citations with an annual average of 1,766 citations, and have received more than 3,500 citations in each of the past 5 years.

### Visual analysis of countries and institutions

**Figure 3:**
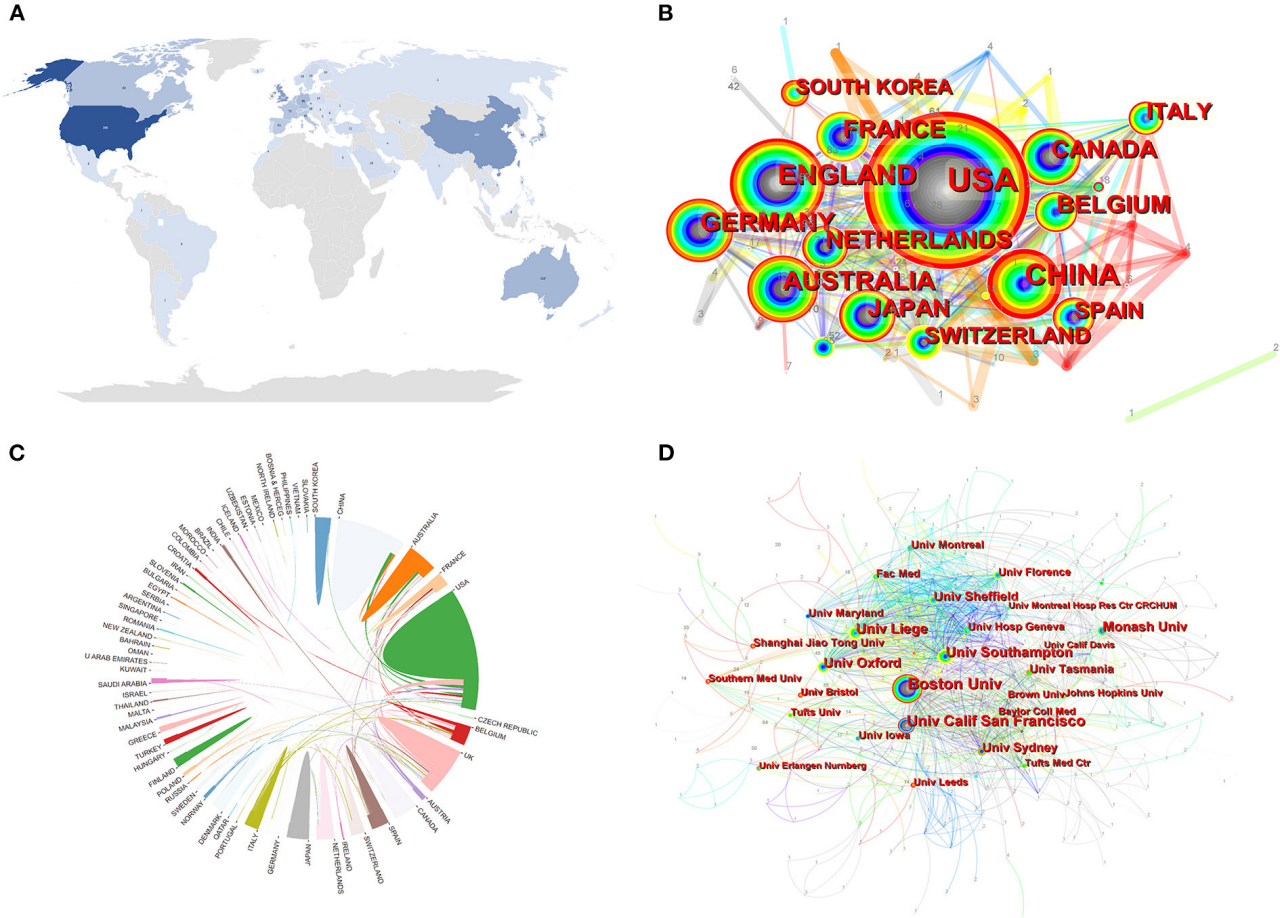
**(A)** A world map depicting the contribution of each country based on publication counts. **(B)** Spatial distribution map of countries/regions. **(C)** International collaboration analysis among different countries. **(D)** Spatial distribution map of institutions.

thousand five hundred twenty six academic institutions from 66 countries have published papers in the field of research on the relationship between OA and OP. As we could see in the Table 2, among them, The United States Published the most papers in this field, with 350 publications, accounting for 32.47% of the total, followed by China (184, 17.07%), the United Kingdom 162 (15.03%), and Australia (110, 10.20%). As for other countries, the publications are all under 100. Figure 3A shown very visually the distribution of all oa and op publications around the world from 1998 to 2021, with darker blue representing more publications. We could see that most papers are published by researchers from developed countries such as North America and Western Europe. Figures 3B,C shown the international cooperation among various countries and regions. The thickness of the link between the two countries indicates the strength of the cooperation. It is not hard to find the closest cooperation between the United States and the United Kingdom, South Korea, China, and Australia. In addition, Boston University was the academic institution that published the most papers (85, 7.885%), followed by the University of California, San Francisco (68, 6.308%) and the University of Liege (46 Articles, 4.267%). It is clear that the top two institutions are all in the United States, and we can see in Table 2 that the world's top ten output institutions in this field are in the United States, and the rest are in developed countries such as Europe and Oceania. In addition, some countries and research institutions, such as the United States (0.25), England (0.27), Belgium (0.24), Germany (0.14), Australia (0.10), and Boston University (0.20), showed high centrality, circled in purple in Figures 3B,D.

**Table 2:** Publications in the 10 most productive countries/regions and institutions.

**Rank**	**Country**	**Year**	**Counts (%)**	**Centrality**	**Institutions**	**Year**	**Counts (%)**	**Centrality**
1	USA	1998	350 (32.47)	0.25	Boston University	2000	85 (7.88)	0.20
2	China	1998	184 (17.07)	0.04	University of California San Francisco	1999	64 (5.94)	0.09
3	England	1998	162 (15.03)	0.27	University of Southampton	2011	45 (4.17)	0.05
4	Australia	1998	110 (10.2)	0.10	University of Liege	2012	44 (4.08)	0.06
5	Germany	2003	95 (8.81)	0.14	University of Oxford	2009	39 (3.62)	0.08
6	Canada	2004	83 (7.70)	0.07	Monash University	2004	35 (3.25)	0.04
7	French	2000	73 (6.77)	0.04	University of Sydney	1999	30 (2.78)	0.08
8	Netherlands	1999	71 (6.59)	0.10	University of Sheffield	2013	27 (2.50)	0.04
9	Japan	1998	70 (6.49)	0.00	University of Tasmania	2007	24 (2.23)	0.01
10	Belgium	2000	68 (6.31)	0.24	University of Montreal	2006	20 (1.86)	0.05

### Analysis of funding organizations

**Figure 4:**
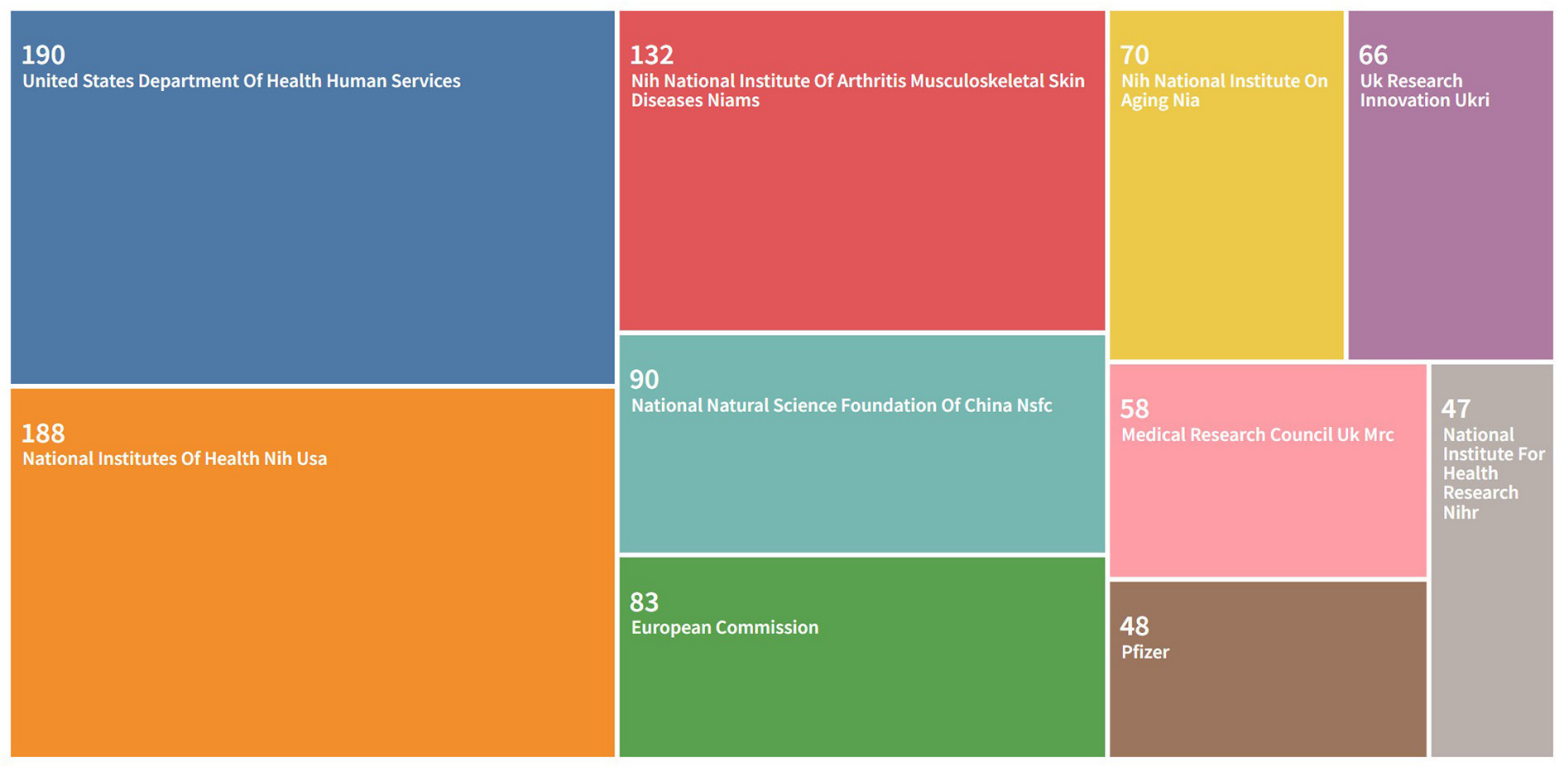
Top 10 funding organizations.

Figure 4 showed the top 10 funding organizations for research on the relationship between OA and OP. According to the distribution of funding organizations, six of them are from the United States, while the other four are from the European Union, China, and the United Kingdom. According to the information, The U.S. Department of Health and Human Services (HHS) funded the largest number of research projects, 190. The National Institutes of Health (NIH) and the National Institute of Musculoskeletal Skin Diseases of Arthritis (NIAMS) ranked second and third with 188 and 132 studies, respectively.

### Analysis of the authors and co-cited authors

**Figure 5:**
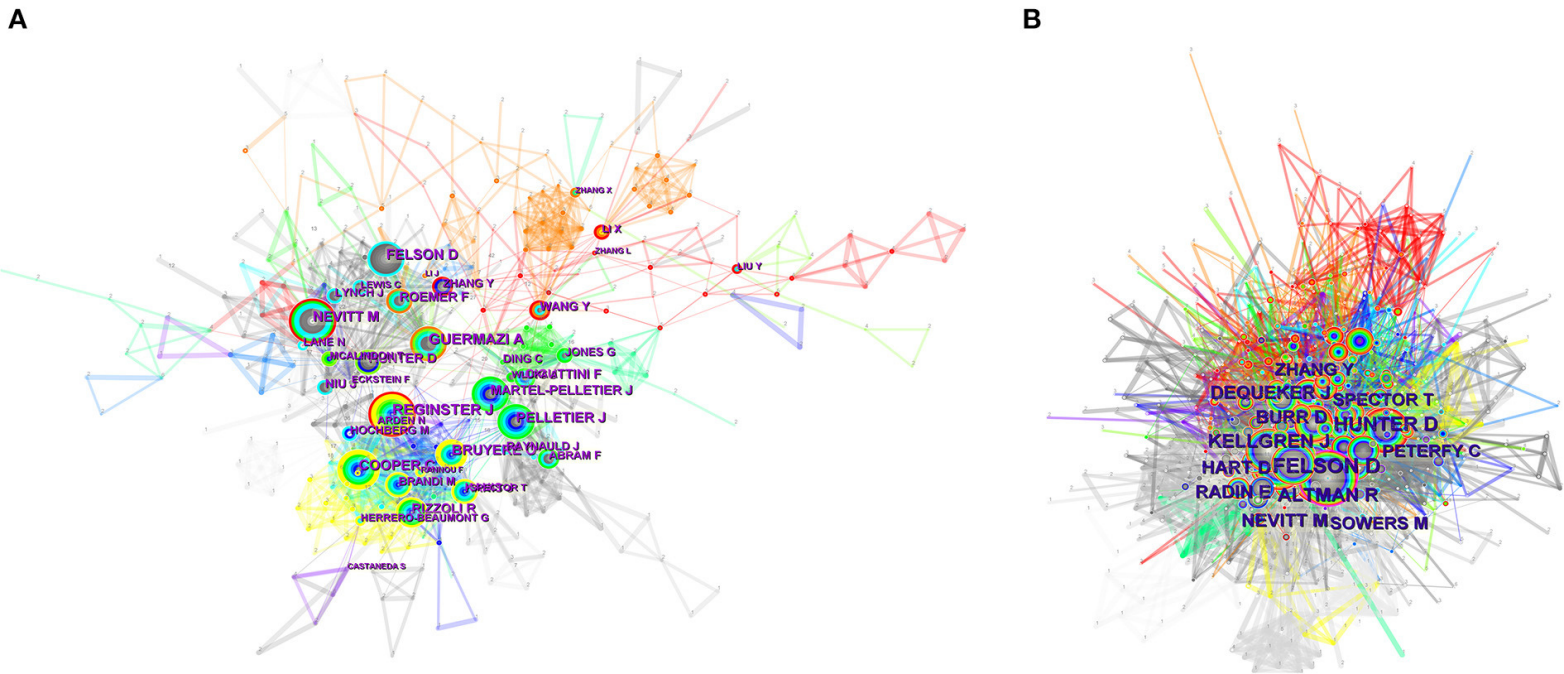
**(A)** Visual analysis of authors. **(B)** Visual analysis of co-cited authors.

Scholars working in different fields have unique perspectives in their respective fields, and collaboration in this area can facilitate communication between disciplines and unleash new productivity. In addition, the analysis of co-authors helps other scholars understand existing partnerships. The same discovery of relevant contributions by influential researchers can provide authoritative information on certain research areas to help other researchers discover new directions and guidelines. 5527 authors and 19,163 co-cited authors were associated with the relationship between OA and OP. Table 3 listed the top 10 authors by number of published articles. The top 10 authors in the field of OA and OP relationship published 450 publications, accounting for 41.74%, or nearly half of the total. Reginster JY from Belgium was the most published author, followed by Guermazi A, and Cooper C. But we should note that the betweenness centrality is relatively low and were all <0.1. These revealed that the authors' work is very little related to each other and does not have much influence on each other. As shown in Figure 5A, on the visualization of author collaboration.

**Table 3:** Top 10 authors and co-cited authors.

**Rank**	**Author**	**Year**	**Counts**	**Centrality**	**Co-cited author**	**Year**	**Counts**	**Centrality**
1	Reginster JY	2007	46	0.03	Felson DT	1999	276	0.04
2	Guermazi A	2004	43	0.03	Hunter DJ	2005	201	0.04
3	Cooper C	2011	42	0.02	Altman R	1998	182	0.11
	Felson DT	2000	42	0.01				
4	Nevitt MC	1999	37	0.05	Kellgren J	1998	180	0.08
5	Pelletier JP	2006	36	0.05	Burr D	1998	144	0.06
6	Bruyere O	2003	35	0.05	Dequeker J	1998	136	0.06
7	Cicuttinif F	2004	30	0.03	Hart D	1998	127	0.05
	Martel Pelletier J	2007	30	0.03				
8	Rizzoli R	2013	29	0.01	Nevitt MC	1998	123	0.03
	Hunter DJ	2006	29	0.12				
9	Roemer F	2009	27	0.02	Radin E	1998	121	0.07
10	Kanis J	2013	24	0.00	Zhang Y	2002	110	0.05

Co-citation analysis is an important part of bibliometrics, which refers to two authors/literature works that appear simultaneously in the reference list of the third literature (32). Co-cited authorship means a specific relationship between the authors of two or more articles cited simultaneously by another paper or papers. Therefore, author co-citation analysis generally reveals the key author of co-citation networks in the field. In general, authors who are cited more often are considered to have more influence. As can be seen from Table 3, among the top 10 co-cited authors, all of them were cited more than 100 times. Felson DT was the most frequently co-cited author, cited 276 times, followed by Hunter DJ (201), Altman R (182), and Kellgren J (180). We can see that Altman R has a high Centrality (0.11). As Figures 5A,B indicated that there is a communication and cooperation network between authors and co-cited authors in this research field.

### Analysis of journals and co-cited journals

**Figure 6:**
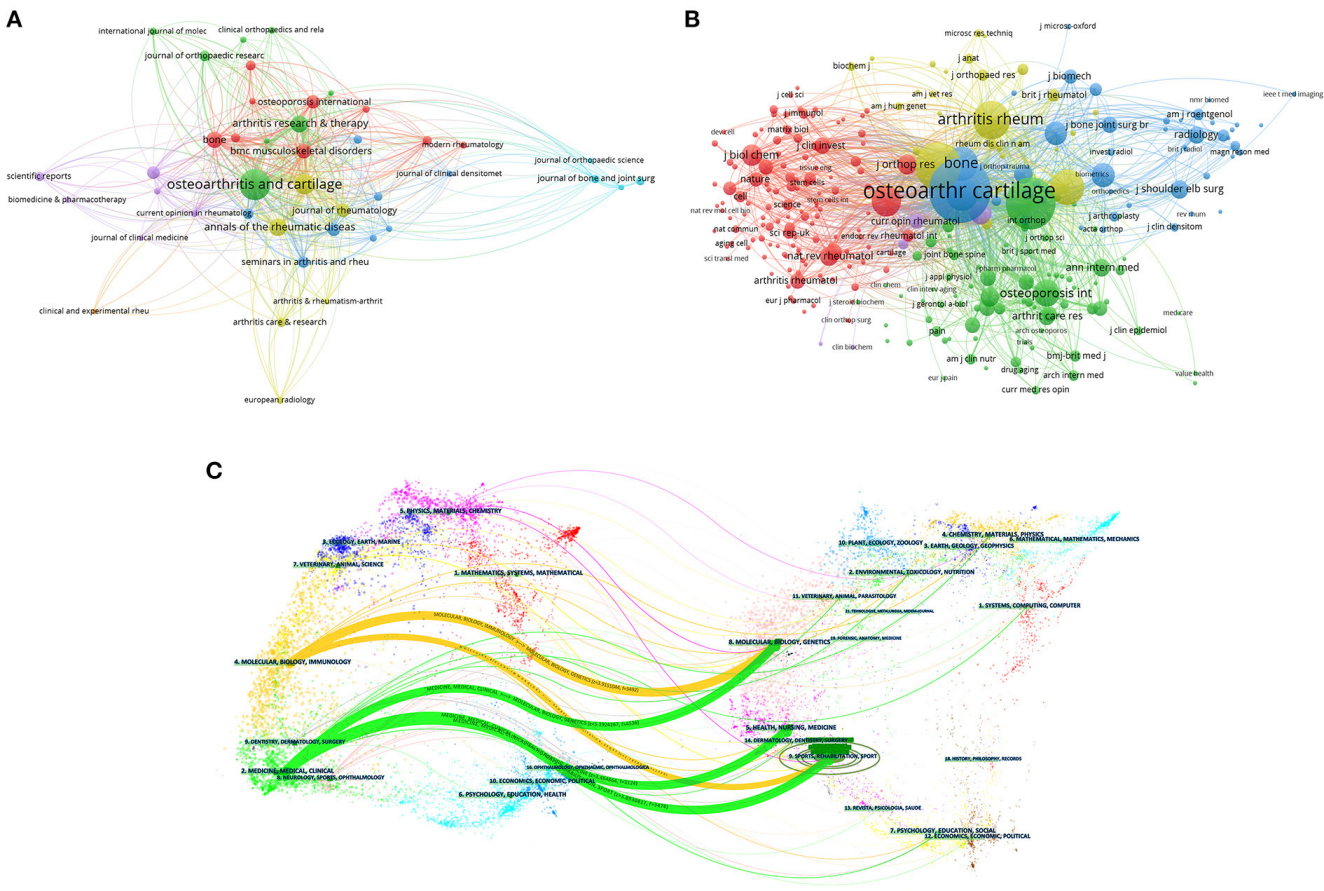
**(A)** Network visualization map of journal. **(B)** Network visualization map of Co-Cited Journals. **(C)** The dual-map overlay of journals. The citing journals were on the left, the cited journals were on the right, and the colored path represents the citation relationship.

For a long time, journals have been an important platform for scientific communication among scholars in various fields. Therefore, the analysis of journal source distribution helps researchers to find the most suitable journals for their articles in a short period. According to preliminary statistics, a total of 318 journals have publications that study the relationship between OA and OP. Table 4 summarized some basic information about the top ten journals by several publications. Among them, Osteoarthritis and Cartilage (127/11.78%) produced the largest amount. This was followed closely by Arthritis and Rheumatism (54/5.01%), Annals Of The Rheumatic Diseases (41/3.80%), and Arthritis Research Therapy (39/3.62%). In addition, JIF is data from Journal Citation Reports (JCR) produced by Thomson Reuters. It is an internationally recognized index for journal evaluation. Among the top 10 academic journals, Annals of the Rheumatic Diseases (27.973) has the highest JIF, followed by Arthritis Rheumatology (15.483), and Osteoarthritis and Cartilage (7.507). According to the classification of each discipline, according to the influence factor of the journal in the current year, the journal is divided into four areas on average, which are Q1, Q2, Q3, and Q4, each accounting for 25%. As can be seen in Table 4, 50% of the journals belong to Q1. This indicates that there is a strong tendency for publications published in this field to be listed in the top issue. Among the 4,328 co-cited journals, seven journals had more than 1,000 citations, As presented in Table 5, Osteoarthritis And Cartilage had the most co-citations (citations:4425, IF: 7.507), followed by Annals Of The Rheumatic Diseases, and Arthritis Rheumatology-US. According to the 2021 Journal citation reports (JCR), The visualizations of journals and co-cited journals can be seen in Figures 6A,B, respectively. Figure 6C shows the dual map overlay concerning OA and OP published between 1998 and 2021.

**Table 4:** Top 10 journals with the most publications.

**Rank**	**Journal**	**Counts**	**% of 1,078**	**JIF (2021)**	**Quartile in category (2021)**
1	Osteoarthritis and Cartilage	127	11.78	7.507	Q1
2	Arthritis and Rheumatism	54	5.01	0	-
3	Annals of the Rheumatic Diseases	41	3.8	27.973	Q1
4	Arthritis Research Therapy	39	3.62	5.606	Q1
5	Bone	35	3.25	4.626	Q2
6	BMC Musculoskeletal Disorders	29	2.69	2.562	Q3
7	Osteoporosis International	25	2.32	5.072	Q2
8	Journal of Rheumatology	23	2.13	5.346	Q2
9	Arthritis Rheumatology	22	2.04	15.483	Q1
10	Rheumatology	21	1.95	7.046	Q1

**Table 5:** Top 10 co-cited journal.

**Rank**	**Co-cited journal**	**Citations**	**JIF (2022)**	**Quartile in**
				**category (2022)**
1	Osteoarthritis and Cartilage	4,425	7.507	Q1
2	Annals of the Rheumatic Diseases	2,879	27.973	Q1
3	Arthritis Rheumatology-US	2,414	15.483	Q1
4	Arthritis Rheumatology	1,572	0	-
5	Journal of Rheumatology	1,344	5,346	Q2
6	Bone	1,244	4.626	Q2
7	Journal of Bone and Mineral Research	1,169	6.39	Q1
8	Arthritis Research Therapy	996	5.606	Q2
9	Osteoporosis International	772	5.072	Q1
10	Journal of Bone and Joint Surgery-American Volume	619	6.558	Q1

### Visual analysis of co-citation and clustering network

**Figure 7:**
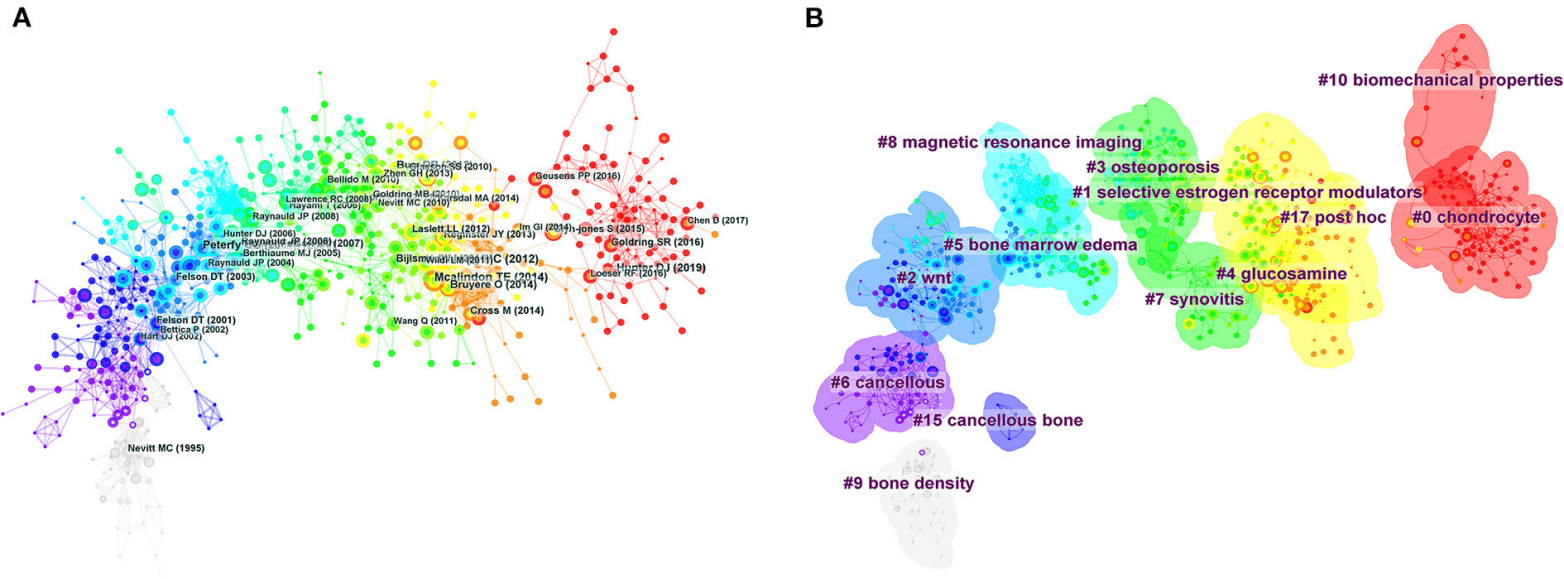
**(A)** Visual analysis of co-citation. **(B)** Clustering network.

In 1973, Small and Mashkova proposed that co-citation analysis should be used as a way to assess links between articles (33, 34). It is generally accepted that if several publications are cited by later publications at the same time, the cited paper is defined as having a co-citation relationship (35). Citations and co-citation analysis are the core functions of CiteSpace (15). Figure 7A shown the years of co-citation, first author, and top 10 most cited articles for 29,254 articles. The more information about the top 10 references shown in Table 6. The most co-cited reference performed by Kellgren JH et al., was an original article published in Annals Of The Rheumatic Diseases, entitled“Radiological assessment of osteoarthrosis,” followed by an article entitled“Role of subchondral bone in the initiation and progression of cartilage damage.” The largest clusters extracted from the references of 1,078 cited articles are shown in Figure 7B.

**Table 6:** Top 10 co-cited references.

**Rank**	**Title**	**Journals**	**First author**	**Co-citations**	**References**
1	Radiological assessment of osteoarthrosis	Annals of the Rheumatic Diseases	Kellgren JH	174	(36)
2	Role of subchondral bone in the initiation and progression of cartilage damage	Clinical Orthopedics Related Research	Radin	98	(37)
3	Radiographic osteoarthritis of the hip and bone mineral density. The Study of Osteoporotic Fractures Research Group	Arthritis Rheumatology-US	Nevitt MC	86	(38)
4	Development of criteria for the classification and reporting of osteoarthritis. Classification of osteoarthritis of the knee. Diagnostic and Therapeutic Criteria Committee of the American Rheumatism Association	Arthritis Rheumatology	Altman R	82	(39)
5	The relationship between osteoarthritis and osteoporosis in the general population: the Chingford Study	Annals of the Rheumatic Diseases	Hart DJ	75	(7)
6	Whole-Organ Magnetic Resonance Imaging Score (WORMS) of the knee in osteoarthritis	Osteoarthritis and Cartilage	Peterfy CG	75	(40)
7	Validation study of WOMAC: a health status instrument for measuring clinically important patient-relevant outcomes to antirheumatic drug therapy in patients with osteoarthritis of the hip or knee	Journal of Rheumatology	Bellany N	74	(41)
8	Association of radiographically evident osteoarthritis with higher bone mineral density and increased bone loss with age. The Rotterdam Study	Arthritis Rheumatology	Burger H	69	(42)
9	Bone density, osteoarthrosis of the hip, and fracture of the upper end of the femur	Annals of the Rheumatic Diseases	Foss MVL	67	(43)
10	The role of subchondral bone remodeling in osteoarthritis: reduction of cartilage degeneration and prevention of osteophyte formation by alendronate in the rat anterior cruciate ligament transection model	Arthritis Rheumatology-US	Havami T	67	(44)

### Visual analysis of keyword co-occurrence

**Figure 8:**
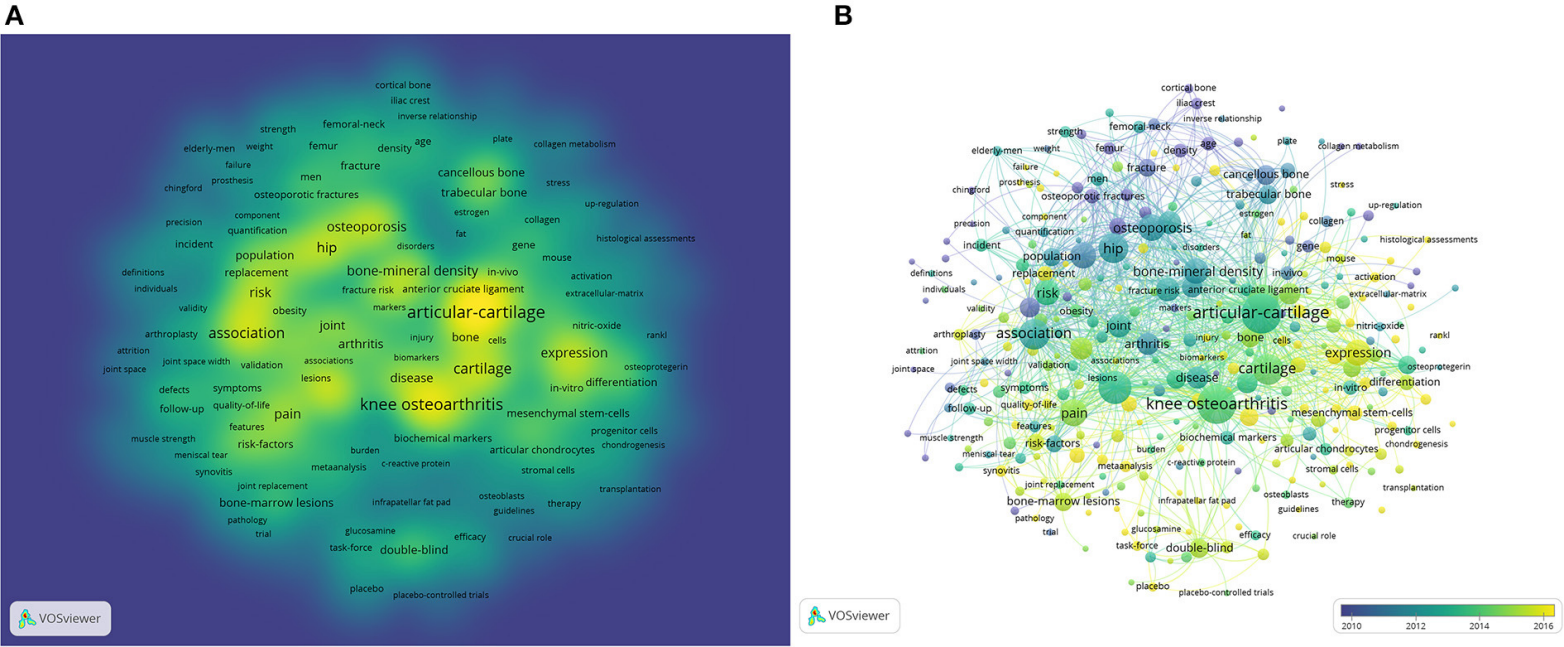
**(A)** The density visualization map of keyword co-occurrence analysis. **(B)** Visual analysis of keyword co-occurrence.

In the study of scientific knowledge structure, keyword co-occurrence was an efficient bibliometric analysis method, because it can relatively accurately capture the hot areas. We used VOSviewer to extract keywords from the titles and abstracts of 1,078 papers for analysis, and 2,495 keywords were extracted, among which 167 keywords appeared more than 10 times and 22 keywords appeared more than 50 times. As we can see from Table 7, articular cartilage was the most important term with 201 co-occurrences, followed by knee OA, progression, cartilage, and association. In the keyword co-occurrence visualization, keywords are colored differently according to their average publication year. This is shown in the density visualization in Figure 8A, several clusters of hotspots related to knee OA, articular-cartilage, association, and OP were observed. Among them, OA, fracture, fracture, and loose fracture appear in the early stage, which could be seen in Figure 8B.

**Table 7:** The top 20 keywords.

**Rank**	**Keywords**	**Occurrences**	**Total link strength**	**Rank**	**Keywords**	**Occurrences**	**Total link strength**
1	Articular-cartilage	201	1,077	11	Women	85	526
2	Knee osteoarthritis	190	1,106	12	Bone-mineral density	80	460
3	Progression	139	834	13	Risk	80	462
4	Cartilage	119	611	14	Knee	73	372
5	Association	115	719	15	Joint	69	361
6	Hip	113	685	16	Mineral density	66	384
7	Subchondral bone	113	686	17	Arthritis	62	356
8	Osteoporosis	92	577	18	Disease	61	328
9	Pain	89	484	19	Prevalence	61	349
10	Expression	87	449	20	Model	57	316

## Discussion

In this study, we analyzed the research field of the relationship between OA and OP through bibliometrics and visualization, and comprehensively understood the change of the global research trend in this field, including countries, institutions, funding institutions, journals, authors and keywords. It is clear that the field of study has grown rapidly over the past two decades, with the number of publications increasing year by year. We retrieved 1,078 articles in WoSCC according to the designed retrieval strategy. Based on currently available information, we can infer that, as the ceaseless improvement of living levels, the increase of the average life expectancy of the world's population and in turn affected the population increase of OA (45), that at the same time because the OP is a major clinical problem of elderly men and women, so the increase of the aging population, also caused the diseased population increase (46), the disease mechanism, Treatment has also become the major public health issue of this century. Based on currently available information, we can infer that, as the ceaseless improvement of living level, the increase of the average life expectancy of the world's population and in turn affected the population increased, OA that at the same time because the OP is a major clinical problem of elderly men and women, so the increase of the aging population, also caused the diseased population increase, the disease mechanism, Treatment has also become the major public health issue of this century. This is an important factor in the rapid growth of publications on the relationship between OA and OP since 1998.

As for the spatial distribution of countries and institutions, the United States, China, the United Kingdom, and Australia have played a major role in the study of the relationship between OA and OP. These four countries are the top four most prolific countries, producing more than half of all publications and leading the rest of the world. Among them, the United States, China, the United Kingdom, Australia, and Japan were the first countries to do research in this field, followed by the Netherlands, France, and Belgium. The number of articles published in Western Europe accounted for more than half of the total, 1.41 times that of North America, 2.08 times that of East Asia, and 5.37 times that of Oceania. These data indicate that Western Europe is the most active region in this field. But separately at the national level, the United States has been particularly prolific. In my opinion, the USA as the only superpower in the world has superior conditions in basic and clinical trials, and is ahead of other countries in terms of science and technology, with its professional technology and equipment, full-time researchers, and sufficient scientific research funds, so that it firmly occupies the leading position in the field of research. Of course, as the economies of Asian and European countries continue to grow, interest in this area of research increases, and research led by these countries increases, the gap with the United States will gradually narrow. In 1978, Freeman, an American sociologist, and professor, first proposed intersex centrality as an index to measure the degree to which a point is located in the “middle” of other “point pairs” in the graph (47). It is mainly used to measure the value of the bridging function of nodes in the whole network structure (48). As can be seen from Table 2, the United States, the United Kingdom, Germany, and Belgium has a high centrality (>0.1). However, other countries are less intermediate than the above four countries. This result may be the relative lack of cooperation between these leading countries and other countries, and the large number of papers published alone, resulting in low centrality. By and large, most partnerships have been limited to the better-off economies of Europe, the U.S., and East Asia. In the economy that was backward, less developed countries have little cooperation and need to be strengthened more. Most of these institutions are located in developed countries, and it is found from the inter-institutional cooperation graph that most of the cooperation is in European and American countries, and only Boston University has a centrality > 0.1. The lack of international cooperation may significantly reduce the flexibility of research efficiency. The quality of research could be further improved by extensive inter-institutional exchanges.

In the visualization analysis of funding agencies, Figure 4 shown the top 10 funding agencies, of which 6 are from the US and the remaining 4 are from the EU, China and the UK. The United States is a leader in the field of research on the relationship between OA and OP, and to maintain this advantage, adequate financial support is essential.

For the analysis of the authors with the largest contribution in the field of OA and OP, we ranked the number of published publications and evaluated them in combination with other indicators to provide a more comprehensive perspective on the contribution of these authors (49, 50). Reginster JY (46 publications) was the most prolific author, followed by Guermazi A (51), and Cooper C (52). Reginster JY and his team found through research in 2003 that the width of medial knee joint space is closely related to tibial subchondral bone mineral density (BMD). The larger the tibial subchondral bone mineral density, the smaller the width of medial knee joint space, showing a high negative correlation (52). These suggested that assessment of subchondral BMD in the medial tibial plateau may be a good predictor of the degree of medial space stenosis in knee OA. On the other hand, we must note that. Then Cooper C teamed up with Reginster JY and conducted a randomized controlled trial of the efficacy and safety of an oral anti-OP drug, strontium ranelate, in the treatment of knee OA (51). They founded that using strontium ranelate in OP patients not only reduced bone loss but also isolated bone remodeling. It can inhibit subchondral bone resorption and stimulate cartilage matrix formation *in vitro*. Strontium ranelate reduces the expression of biomarkers of cartilage degradation, thereby reducing the progression and clinical symptoms of spinal OA, which revealed the potential of strontium ranelate in the treatment of knee OA. Reginster JY's greatest contribution is a very detailed review of OA in disease management (53, 54), the dangers of disease (55), treatment of disease (56–59), and the value of molecular markers (60), carried out a relatively comprehensive summary. Meanwhile, a lot of work has been done on the diagnosis (61, 62), management (63), evaluation and treatment of OP (64–66). The above is of great significance for other scholars to discover the relationship between OA and OP. While the main contribution of professor Guermazi A was the role of radiological techniques in the assessment of OA (67–69). But as shown in Figure 5A, these top three published authors do not form an obvious network trend among each other, and the centrality is relatively low and were all <0.1, which indicates that they have little contact with each other, their work has very little influence on each other, and they do not carry out excessive academic exchanges. In addition, two authors were cited more than 200 times, Felson DT received the most co-citations (276), followed by Hunter DJ (201) but their centrality is also at a low level. In view of the low level of centrality between authors and co-cited authors, we should advocate that many scholars in various countries and regions should carry out more academic activities, strengthen cooperation and communication between each other, and push the research field of the relationship between OA and OP to a new height.

Additionally, the analysis of journals and co-citations can provide information about the journals which are friendly to certain fields of study (70). This information can be used by researchers to select appropriate journals for publication (71). Publishing research results in a journal of considerable international influence an important part of establishing effective academic exchanges (72, 73). Analyzing journal sources helps researchers quickly find the best journals for their articles. Table 4 showed that Osteoarthritis And Cartilage (127 publications, IF: 7.507) published the most articles. In addition to the number of papers, journals are judged on how often they are co-cited in research. Coincidentally, Osteoarthritis And Cartilage also attracted the most co-citations as shown in Table 5. Followed by Annals of The Rheumatic Diseases, Arthritis Rheumatology-US. The results shown that these journals publish most of the high-quality research, attract a lot of attention from scholars interested in the field and provide a considerable boost to their respective work. In the WoSCC database, we analyzed the retrieval results. The top 10 subject categories in terms of the number of publications are shown in Table 6. Rheumatology, Orthopedics, and Endocrinology Metabolism is the discipline that attracts the most attention in this field. In the dual-map overlays, the original map for citations was generated from over 10,000 Journals Indexed In WoSS (74). In our study, the research data related to the relationship between OA and OP were imported into CiteSpace to make the dual map coverage of corresponding journals, and the subject distribution of journals involved in this field was visualized. Through this method, we can intuitively find the flow trend of information in this field in different disciplines and identify the relevant subject hotspots. As presented in Figure 6C, the coarsest striped representation shows the five core citation paths. The orange paths indicated publications in molecular/biological/immunology journals that study the relationship between OA and OP, usually cited in journals on molecular/biological/Genetics and Sports/Rehabilitation/Sport. The green paths implied that the majority of publications which published in the journals of Medicine/Medical/Clinical are likely to be biased to cite papers published in journals on Sports/Rehabilitation/Sport, Health/Nursing/Medicine, and Molecular/Biology/Genetics. Meanwhile, the IF>5 accounted for most of the top 10 journals (80%) and the co-cited journals (80%), indicated that these journals play an important role in this field and suggesting from another perspective that researchers interested in this field should pay more attention to these journals.

The visual analysis of co-citation can be seen in Figure 7, each circle represents a reference. The diameter of the circle is proportional to the number of citations. The association between the two circles represents two articles cited by the same article among the 1,078 articles (cited articles) retrieved in this study. Similarly, the thickness of the lines was positively correlated with the number of co-citations, as shown in Figure 7A. In addition, cluster analysis can show the knowledge structure of the research field. Through the analysis of co-cited literature and clustering, the research topics in specific fields are summarized. In CiteSpace, two concepts of modularity value (Q value) and average silhouette value (S value) are proposed based on the definition of network structure and clustering. They can serve as the basis for judging the drawing effect of atlas. Q value is generally in the interval (0,1), when it is larger than 0.3, it means that the divided community structure is significant; for S value, if it is larger than 0.5, it is considered that the clustering is reasonable; if it is larger than 0.7, it is considered that such clustering is convincing (72, 75–77). In our study, the Q value was 0.8074, which indicated the rationality of the network. The mean S value was 0.9324, and the S values of clusters #0-#17 are all > 0.8, indicating that these clusters have good homogeneity. Figure 7B showed “chondrocyte” was the largest cluster (#0), followed by “selective estrogen receptor modulators”(#1), and “wnt” (#2). Considering the reasons for the formation of clustering phenomenon, we believe that the early studies of diseases mainly focus on the specific changes of diseases. For example, one of the pathological characteristics of OA is cartilage wear, and cartilage wear is usually closely related to sports injuries. Therefore, OA has been extensively studied in this field. On the other hand, OP as a bone metabolic disease, combined with the high prevalence of OP in postmenopausal women, selective estrogen receptor modulators are closely associated with this aspect (78), selective estrogen receptor modulators prevent extensive bone loss by increasing trabecular bone mineral density. The serum levels of IL-6 and cartilage destruction markers were decreased. Moreover, there is a corresponding strong relationship between selective estrogen receptor modulators and OA (79, 80), they showed that estrogen can affect the activity of joint tissues in many ways through complex molecular pathways. In addition, estrogen replacement therapy and current selective estrogen receptor modulators have synergistic effects in maintaining or restoring joint tissue in OA. Finally, with the in-depth study of disease, more and more evidence has found that OA and OP are related, and some studies have found that WNT signaling pathway plays an important role in bone growth and development. Many polymorphisms in this pathway are associated with OA/OP. Increased WNT signaling activity strengthens bone but may have adverse effects on articular cartilage (81, 82).

In this study, 2,495 keywords were extracted from all publications and analyzed by VOSviewer. Through the extraction and analysis of keywords, we intuitively conclude from the visualization results that keywords have diversity and variability over time. By analyzing their essence from the surface phenomenon, keyword diversity reflects the complexity of the disease mechanism. Single keywords cannot perfectly summarize the relationship of OA and OP, which indicates that the connection between them is not the connection between a single disease, and they may affect each other and form cause and effect in different development processes through various mechanisms. Mostly we found early keywords at the same time around the disease itself, from a macro level analysis of the relationship between the two, while the late keywords more and more attention to the root cause of the disease-related information, it shows that with the deepening of the research, we are no longer mere attention to appearances, gradually will focus on inflammation causes, inflammatory factors and cellular and molecular levels. As observed in Figure 8B, in the keyword co-occurrence visualization, author keywords are colored differently according to their average publication year. Among them, OA, fracture, and osteoporotic fracture appear in the early stage. In a study of femoral neck fractures, OA and OP in the middle of the last century (83), OA and OP were found to be almost not etiologically related in patients with femoral neck fractures, but both could eventually lead to injury, it is not difficult to find that, at this stage, the relationship between multiple parties only stops at one side inducing the emergence of the other. With the change of time, researchers began to focus on disease not only on the symptoms of disease, but more deeply into its essence, focusing on the causes of inflammation, inflammatory factors, and cellular and molecular levels. Researchers have focused more on key words such as inflammation, expression, and mesenchymal stem cells and other keywords are highlighted in yellow, indicating that these fields have attracted more attention in recent years and are hot areas of research, in recent years, there has been much discussion about the role of inflammation in OA and OP. Some plant extracts can prevent bone loss in OA and OP models by inhibiting inflammation and have been demonstrated in animal models (84). With the progress of medical technology, gene research has gradually become a research hotspot. Darja et al. founded that the expression of MMP-9 and osteocalcin in proximal femur of osteoarthritis is higher than that of femoral fracture, indicating that bone remodeling of osteoarthritic bone is increased. In addition, RANCL-OPG, an enzyme involved in bone resorption, differs in the regulation of osteoarthritic and osteoporotic bone (85). At the molecular level, other studies have shown that the expression of osteoclast and anti-osteoclast proinflammatory cytokines is different in human OP and OA (86), which may be an important factor in the bone phenotypic characteristics of OP and OA. For mesenchymal stem cells, the effect of mesenchymal stem cells on femoral fracture healing in OA and OP patients has also been explained in the literature (87). From the changes of these hot keywords over time, we find that the research on the relationship between OA and OP follows an obvious rule. From the mutual influence of the first two diseases themselves, to the study of their etiology, to the study of the treatment of the two diseases. Therefore, it is not difficult to infer that in the future, the development of medical technology will surely bring about the improvement of the level of diagnosis and treatment of diseases, and the research on the relationship between OA and OP will focus on the reform of new technologies and new treatment methods when the pathological mechanism of diseases is clear. Bibliometrics is based on several empirical statistical laws, such as Lotka's law, which represents the distribution of authors in scientific and technological literature; Zipf's law to characterize the distribution of word frequency in literature and Bradford's law to determine the distribution of a subject's papers in journals. Bibliometrics has been developing around these laws in two directions: one is to verify and improve these empirical laws; The other is to expand and popularize the practical application of these empirical laws. In conclusion, when studying the relationship between OA and OP, we also mentioned the contribution of countries, institutions and authors, mainly for the following purposes: (1). To identify the classical literature and knowledge foundation in the research field; (2). Show the evolution of subject knowledge in different time periods; (3). Explore research hotspots and development trends of various disciplines; (4). Reveal the major contributors to a research field (countries, institutions, scholars, etc.); (5). Help researchers acquire the context of a knowledge field in a comprehensive, scientific and rapid manner. We think this is a necessary and profound area of research.

## Limitation

Through visual analysis of bibliometrics, the structure and temporal dynamics of this field can be understood to some extent, but this study also has some limitations that are difficult to eliminate. First, the data set we included only includes data from the WoSCC database, which may leave out some relevant studies from other large databases. However, as the most used dataset in the field of bibliometrics, WoSSC also has sufficient data to illustrate the current status of the research field on the relationship between OA and OP. Second, Second, we excluded non-English language literature, so some contributions to the field using non-English language regions were overlooked. Third, since we set the search strategy until 2021 and the database is constantly updated, the impact of recent publications on the field may be underestimated

## Conclusion

With the help of CiteSpace and VOSviewer, we have a deeper understanding of the current status, cutting-edge hotspots, and future trends of the research on the relationship between OA and OP during 1998–2021. The United States and China are in the leading position. However, there is a lack of cooperation and communication among countries, institutions, and authors, and it is necessary to strengthen the cooperation and communication among them. According to the time variation of hot spots, inflammation, expression, mesenchymal stem cells will be the focus of future studies on the relationship between OA and OP.

## Data availability statement

The raw data supporting the conclusions of this article will be made available by the authors, without undue reservation.

## Author contributions

XWan and XWang designed, data analysis, interpretation, and manuscript writing. RP, CX, and WS collected the data. HZ, ZL, and HL revised and approved the final version of the manuscript. All authors contributed to the article and approved the submitted version.
